# A Qualitative Study of Views and Experiences of Women and Health Care Professionals about Free Maternal Vaccinations Administered at Community Pharmacies

**DOI:** 10.3390/vaccines8020152

**Published:** 2020-03-29

**Authors:** Natalie Gauld, Samuel Martin, Owen Sinclair, Helen Petousis-Harris, Felicity Dumble, Cameron C. Grant

**Affiliations:** 1 Department of Paediatrics: Child and Youth Health, University of Auckland, 2 Park Rd, Auckland 1023, New Zealand; cc.grant@auckland.ac.nz; 2 Huntly West Pharmacy, Huntly 3700, New Zealand; samuel.martin1707@gmail.com; 3 Waitematā Hospital, Auckland 0610, New Zealand; Owen.Sinclair@waitematadhb.govt.nz; 4 Department of General Practice and Primary Health Care, University of Auckland, Auckland 1023, New Zealand; h.petousis-harris@auckland.ac.nz; 5 Waikato District Health Board, Hamilton 3204, New Zealand; Felicity.Dumble@waikatodhb.health.nz; 6 General Paediatrics, Starship Children’s Hospital, Auckland 1023, New Zealand

**Keywords:** maternal vaccination, community pharmacy, access to medicines, pertussis, influenza, health policy

## Abstract

**Background:** A policy to extend funding of maternal pregnancy influenza and pertussis vaccinations to community pharmacies could address low pregnancy vaccine uptake. The policy has been implemented in one region in New Zealand. This study explored the views and experiences of women eligible for the vaccines and health care professionals regarding funded maternal vaccinations in pharmacy. **Methods:** Women in late pregnancy or with an infant, and midwives, pharmacists, and general practice staff were selected purposively and interviewed regarding maternal vaccinations and the new policy, including their awareness and views of the funded vaccinations in pharmacies, and how this policy worked in practice. Enablers and barriers to vaccination by pharmacists were explored. Interviews were transcribed and analysed using a framework approach. **Results:** Fifty-three interviews were conducted. Most women and health care professionals viewed funded maternal vaccinations in pharmacies positively with respect to increasing awareness and providing delivery options. Many women received messages from pharmacies. Most pharmacies used posters, leaflets and/or verbal explanation to pregnant women to raise awareness of the vaccinations. Not all pharmacies provided these vaccinations, and frontline staff could help to raise awareness. **Conclusion:** Funded maternal vaccinations in pharmacies are generally well accepted and provide an opportunity to increase uptake and prevent disease.

## 1. Introduction

Immunisation during pregnancy is a more recent addition to national immunisation schedules that aims to reduce mortality and morbidity from vaccine-preventable diseases during pregnancy and early infancy. 

Pertussis (whooping cough) causes hospitalisations and deaths, particularly of infants who are too young to have completed their primary series of pertussis vaccination [1,2,3]. In New Zealand (NZ), Māori and Pacific infants are disproportionately affected by pertussis [1], similar to indigenous populations in Australia [4]. A single dose of acellular pertussis vaccine, given during pregnancy as Tdap vaccine (Tetanus, diphtheria and acellular pertussis vaccine), provides protection to this vulnerable age group [5,6,7]. Infant pertussis hospitalisation rates have reduced following the addition of this maternal dose of Tdap to national immunisation schedules [8,9]. 

Influenza infection during pregnancy is associated with adverse fetal outcomes, and with an increased risk of influenza-associated mortality and morbidity for pregnant women compared with the general population [10]. Influenza vaccination in pregnancy is associated with a reduced risk of influenza infection [11,12,13], of influenza-associated hospitalisation of pregnant women [14] and infants [15], and of stillbirth [16]. 

Therefore, maternal vaccinations during pregnancy are recommended in many developed countries [17,18,19]. Health care professional recommendation and/or endorsement of safety encourages uptake of vaccination during pregnancy [20,21,22,23,24,25]. For example, health care professional electronic record automated alerts and the provision of maternal vaccinations by midwives at antenatal appointments significantly increase uptake of a pregnancy dose of acellular pertussis vaccine [26]. However, antenatal vaccine uptake is suboptimal in many countries [22,23,24,27,28,29].

It has been proposed that pharmacy recommendations or funded administration in pharmacy of vaccinations during pregnancy could increase awareness and uptake [21,22,30]. In 2016, the International Federation of Pharmacy (FIP) [31] reported funded pharmacist-administered influenza vaccine during pregnancy occurred in Argentina and the United Kingdom, and, while Belgian pharmacists could not administer vaccinations, they were encouraged to raise awareness of vaccinations to pregnant women and other at-risk groups. The FIP reported that pregnant women were excluded from pharmacist-administered vaccinations in Switzerland. 

Although pharmacy administration of vaccines is associated with increased uptake [32,33], pharmacy administration of vaccinations to pregnant women has received little research to date. A Canadian survey of health care providers of pregnant women found proportionately fewer pharmacists (70%) and midwives (38%) than nurses (84%) and physicians (80%) self-reported recommending the influenza vaccine to all their pregnant patients, however this study was limited by a very low response rate [34]. While not specifically for pregnant women, an intervention in the period 2010–2012 by pharmacists in a US hospital administering Tdap vaccines in the pharmacy or an on-site clinic to close contacts of neonates increased Tdap vaccination of such people from 1.3 per month pre-intervention to 85.2 per month postintervention [35]. Analysis of data from four Canadian pharmacies found 4% of influenza vaccinations administered in the period 2012–2013 were provided to pregnant women [36]. Whether funding administration of maternal vaccinations in pharmacy helps access and uptake and how such a policy is viewed by potential users and pharmacists and other health professionals is unknown. Therefore, a qualitative approach was taken to increase understanding of the effect of the policy to fund maternal vaccinations in pharmacy.

The aim of this study was to explore the effect of funding maternal Tdap and influenza vaccinations through community pharmacies on accessibility and uptake, the awareness and views of health professionals and women of this service, the experience of women in using this service, and barriers and enablers to uptake. This paper presents the qualitative findings related to funded maternal vaccinations during pregnancy in pharmacy. 

## 2. Materials and Methods 

This qualitative study sought a range of views on vaccination during pregnancy, including the funding of these through pharmacy, from users, potential users, and health care providers. The study was conducted in New Zealand with ethical approval granted by the NZ Ministry of Health Northern B Health and Disability Ethics Committee (18/NTB/43). 

### 2.1. Study Setting—New Zealand and Waikato Region

The NZ birth cohort is approximately 60,000 births per year. Uptake of pertussis vaccine during pregnancy has been low in NZ [22], approximately 38% nationally in 2017 (personal communication, Helen Petousis-Harris, 7 February 2020). The most common reason for non-uptake has been lack of awareness in women, with concerns about safety, discouragement by health care professionals and doubt about effectiveness also contributing [20,21]. NZ has universal health care funded from general taxation, providing funding for public hospitals, general practice, maternity care, and pharmaceuticals. Small copayments from the patient are required for general practice and pharmaceuticals but not for public hospitals nor for maternity care through the public system. The NZ government fully funds maternal vaccinations administered in general practice for influenza vaccine at any time in pregnancy and for pertussis vaccine (funded weeks 28–38 gestation at the time of the study) [37]. Women may also be provided fully funded maternal vaccinations in hospitals. Funding for vaccinations are agreed at a national not local level, with the funding at Waikato for this project being extremely unusual. 

In NZ, most women are enrolled with a general practice for primary health care, which includes a free antenatal visit. Women who are not enrolled with a general practice can still attend a general practice (unless the practice is full up) but pay more. However, pregnant women then usually choose a lead maternity carer (LMC) for their pregnancy and birth care and for infant care up to age six weeks, when the first dose of infant vaccinations are due. The LMC is usually a midwife working independently or (less frequently) a hospital midwife, available without charge, and based at a public hospital in one of the 20 District Health Board (DHB) regions in NZ [38]. Women return to a general practitioner for the baby’s six-week check-up and subsequent postnatal and infant care. 

In NZ, maternal doses of pertussis and influenza vaccine during pregnancy have been government funded (completely free for the patient) through general practice and hospitals nationwide since 2010 (influenza) [39] and 2013 (pertussis) [40]. In November 2016, Waikato District Health Board funded pharmacist-administered pertussis vaccine for women at 28–38 weeks’ gestation, and nationally pharmacists became funded from early 2017 to administer influenza vaccine at any time in pregnancy. 

The vaccine administration fee paid by the government to general practice is NZ$21.37 exclusive of goods and services tax (US$13.52), and to pharmacy (for influenza vaccine where there is a national contract) is NZ$19.57 exclusive of tax (US$12.39). The influenza vaccine cost is NZ$9 excluding tax (US$5.70) and the pertussis vaccine price paid by the government is confidential. General practice and pharmacy purchase the influenza vaccine and are reimbursed. General practice is provided with government-paid pertussis vaccine, but for the Waikato project pharmacy is reimbursed. 

Typically, an appointment would be required at the general practice to have the vaccination administered, and general practices are usually only open on weekdays. Pharmacies usually require no appointment for vaccinations, and many pharmacies are open six days a week, with some pharmacies open until late at night, and on Sundays. There is no administration fee charged to the recipient for the vaccination at any venue.

To provide vaccinations in New Zealand, a pharmacist must have successfully completed an approved vaccinator course, been assessed administering two vaccinations, hold a current annual practising certificate, and have a current cardio-pulmonary resuscitation certificate [41]. They must meet the Immunisation Standards for Vaccinators and undergo a two-yearly renewal process. The pharmacy has requirements also, including cold chain requirements and emergency equipment. Legally pharmacies must be at least 51% owned by a pharmacist, who can have a majority interest in up to a maximum of five pharmacies. A minority of pharmacies have corporate shareholdings and over a third of pharmacies operate under a marketing or corporate banner. 

Training and promotion were used to support the delivery of these maternal vaccinations at pharmacies. An educational evening was held for pharmacists in Waikato in October 2016. Each pharmacy (whether vaccinating or not) received two telephone calls with messages about maternal vaccinations, posters promoting free maternal vaccinations in pharmacy and general practice, and a key facts card on vaccinations during pregnancy for staff and could receive t-shirts promoting vaccination during pregnancy. Local consumer media wrote about the initiative. Social media messages were released aimed at female consumers and promoting maternal vaccinations and informing women that free vaccinations during pregnancy were available from pharmacies and general practices. Pharmacies with social media pages were emailed links that they could use to help the campaign. Waikato members of the NZ College of Midwives received two email messages regarding the availability of vaccinations for pregnant women in community pharmacies, including a list of pharmacies offering vaccines. Posters about funded maternal vaccinations in pharmacies and general practices were sent to hospitals and offered to midwives.

Within the Waikato DHB region resides a population of approximately 420,000 people—23% of whom are Māori, 10% Asian, 3% Pacific peoples and 64% of other ethnicities, predominantly NZ European [42]. One-quarter of the Waikato DHB population live in the most deprived quintile of households in NZ [42,43]. There is one city only, Hamilton, and the urban to rural split is 58% to 42% [42]. 

### 2.2. Study Design

Semi-structured interviews were conducted in the Waikato District Health Board area in November 2018 or late January until early May 2019. Eligible participants were from four groups: women who were pregnant or had a young infant (referred to as “women participants”); midwives; community pharmacists; and staff working in general practices. We planned to interview 18–20 women, intending for at least half to be of Māori ethnicity, and 10–12 participants from each of three groups: midwives, community pharmacists and general practice staff. 

### 2.3. Recruitment and Interview Process

Participants from each of the four groups were recruited with the intention to ensure that a wide range of experiences and perspectives and focus particularly on high-need areas and Māori. Half of the Māori women were recruited through the pharmacy the interviewer was working in, and the rest were recruited and interviewed on a Saturday at an extended hours city pharmacy with a large catchment. The other women were recruited through pharmacists and a midwife. We sought to enrol women with variation in age, geographical location, maternal vaccination status, place of vaccination and number of previous pregnancies. All participants needed sufficient English to be interviewed.

Health care providers were selected for variation in their experience and geographical location, focusing most on rural and lower socioeconomic areas, and areas with proportionately larger Māori populations. We sought Māori health care providers, owner and non-owner GPs and pharmacists, and independent midwives and midwives with public hospital employment experience. 

The researcher worked with the College of Midwives regional coordinator to identify a range of midwives who were invited to participate.

For general practice, as practice nurses do most vaccinations, more nurses (all vaccinators) were invited into the study than GPs. Most pharmacists were deliberately selected as those who administered vaccinations, with two owners of rural pharmacies not offering vaccinations also approached. 

Following informed consent, interviews were conducted face-to-face at the participant’s workplace, in their home, in a pharmacy consultation room, or at a café, according to the participant’s preference, or by telephone. A token gift of a NZ$30 supermarket voucher was provided to each participant. The interview topics varied by group, i.e., woman eligible for a vaccination, midwife, pharmacist or general practice staff. For the findings reported in this manuscript, the key topic discussions used were: views and experiences of maternal vaccinations in pharmacy; awareness of maternal vaccinations during pregnancy; barriers and enablers for vaccine access relevant to pharmacy administration; the woman’s journey to having (or not having) a vaccination during pregnancy; barriers and enablers for pharmacy to provide vaccinations during pregnancy; and communication between pharmacy and other health care providers regarding vaccination during pregnancy or vaccination in general. Interviews were audio-recorded (with consent) or notes were taken.

### 2.4. Analysis

Recorded interviews were transcribed verbatim and checked against the recording. Transcripts and notes were read and reread by the first author, then coded using NVIVO Pro. The interviews were coded within four groups: women consumers (identified as Māori or non-Māori); midwives; pharmacists; and general practice staff. Within each group, coding nodes included specific topics discussed (deduction) and emerging themes (induction). For example, under the coding of women consumers were codes such as barriers to getting the vaccine, enablers to getting the vaccine, knowledge, views on pharmacists vaccinating, and the woman’s journey, and themes such as protect baby, empowered, safety, and trust. Analysis took place through a framework approach, by each of the four groups, systematically working through each coding node. Comparisons were made between and within groups for opinions, and experiences, and emerging themes were documented. Findings were shared and discussed between the researchers. 

### 2.5. Researchers’ Roles and Perspectives

Following a training session, the second author, a male Māori pharmacist, interviewed the Māori and Cook Island Māori women using the interview guide, receiving feedback following the first interviews. The first author, a female NZ European pharmacist and experienced interviewer, conducted all other interviews. The first author coded transcripts, conducted analyses and reported findings, with a review from the second author and OS on the Māori women findings before being finalised. 

Both interviewers hold positive views on vaccination but tried not to reveal that stance, rather seeking to understand and encouraging participants to share their views. While pharmacists were aware that their interviewer was also a pharmacist, other health care providers and most women were not. A minority of the Māori women had an existing relationship with the interviewer as their pharmacist. All authors had input into the study and reviewed the findings before being finalised. HPH is a female NZ European vaccination researcher, CCG is a male NZ European academic general paediatrician, OS is a male Māori general paediatrician practising in hospital, and FD is a public health physician. All authors are positive about vaccination. 

## 3. Results

### 3.1. Characteristics of the Study Sample

Nine Māori, one Cook Island Māori and eight non-Māori women participants, 12 pharmacists, 12 people working in general practice, and 11 midwives were interviewed (totaling 53 interviews). Characteristics of the interviewed women are summarised in Table 1 and characteristics of the interviewed health care providers in Table 2.

All 12 pharmacists approached agreed to participate. Ten of the 12 midwives approached agreed to be interviewed, and one additional midwife was recruited through snow balling when the tenth midwife suggested she would provide further useful insight. All seven general practices approached provided a practice nurse, GP and/or practice manager.

Forty-seven interviews were conducted face-to-face, and a further six interviews were by telephone. Most interviews took approximately 25–30 min (range 7–52 min). Two participants declined audio-recording and notes were taken instead. 

The coding of participants indicates their subset with P for pharmacists, M for midwives, N for practice nurse, D for doctor, W for women and MW for Māori or Cook Island Māori women.

### 3.2. Presentation of the Qualitative Data

The qualitative data presented begin with the awareness and views of pharmacists providing funded maternal vaccinations across all of the groups interviewed. It then outlines the uptake of maternal vaccinations in pharmacy and enablers and barriers to pharmacy staff recommending and providing these maternal vaccinations. The results section ends with key themes which included opportunity, and its inter-related themes of convenience, accessibility and proactivity; trust and missed opportunity. Time, awareness and communication also arose often. 

### 3.3. Awareness of Vaccination and Maternal Vaccination in Pharmacy

Most women participants reported that they knew pharmacies provided maternal vaccinations, primarily from pharmacy staff or midwives informing them, and, in one case, by seeing posters in the pharmacy. Three women were unaware of this service in pharmacy, and one was not asked about her awareness of the pharmacy service. 

The vaccinating pharmacists were all aware of funded maternal vaccinations in pharmacy and the time restrictions for funding for Tdap. The non-vaccinating pharmacists were aware of vaccinations in pharmacy but had low awareness of maternal vaccinations. 

All but one midwife was aware of pharmacists offering maternal vaccinations. This awareness arose from a number of sources including information emailed to midwives; their clients or the pharmacy reporting vaccination in pharmacies to the midwife; an immunisation update course; or pharmacy signage. One midwife was informed when receiving her own influenza vaccination in a pharmacy, and two others were contacted by pharmacists telling them about the service being provided. A few midwives did not know which pharmacies provided maternal vaccinations, that this included pertussis vaccine, that there was no cost to the woman, or knew of the service but not that it had started. These midwives tended not to recommend pharmacy as an option for maternal vaccinations.

General practice staff were aware of pharmacists vaccinating, but most were unaware of funded maternal vaccinations or any pertussis vaccine administration in pharmacy. The only report of a general practice staff member informing the participant that their pharmacy provided vaccinations was one nurse. Four general practice participants worked in areas where pharmacies did not provide vaccinations.

### 3.4. Views on Pharmacists Providing Maternal Vaccinations

Women participants were mostly very positive, considering a pharmacy was *“easier”*, and/or noting that they regularly visited pharmacies. Some had qualifications, e.g., providing that they trusted the pharmacy. One participant prone to anxiety preferred a doctor’s office for more reassurance, and another said she did not go to pharmacies generally but would prefer a vaccine from pharmacy.

*“I think if you trust your pharmacist it’s a great idea.”*

W10

All 10 vaccinating pharmacists were very positive about pharmacy provision of vaccinations generally and of funded maternal vaccinations specifically. Mostly they saw convenience for users or opportunity to raise awareness. They also enjoyed the variety and/or patient contact vaccination provided.

*“I think it’s such a valuable service because you’ve got so many people coming in that it frees up medical centres, it frees up nurse and doctor time. So if we can do it and it’s within our scope then why not spread the workload out.”*

P4

One vaccinating pharmacist in a rural high-need area reported negativity from a medical practice about his pharmacy offering vaccinations, including managing adverse reactions and taking their funding, requesting they do not vaccinate patients from their medical practice. This discouraged him from raising vaccinations with consumers. Conversely, one city pharmacist reported *“very positive”* discussions with doctors regarding pharmacy-provided vaccinations, noting the weekend access. 

While positive about vaccinations in pharmacy for access, the two non-vaccinating pharmacy owners outlined multiple barriers, including maintaining their medical practice relationship, and preferring to provide other advanced services rather than vaccination which they saw as a general practice role.

*“…I get the whole demarcation between roles as well, and I still see vaccination in a nursing role.”*

P12

These two pharmacists lacked knowledge about maternal vaccinations, and one worried about safety. They had no pregnancy vaccination posters in their pharmacies, unlike most other pharmacists interviewed. While one reported little need in their rural area because of accessible medical services, the other participant would offer the service if a pharmacist keen on providing vaccinations joined their staff. This pharmacist’s other pharmacy offered vaccinations, but a nearby GP expressed concern about funded influenza vaccination in pharmacy, reportedly having arranged extra general practice staffing for influenza vaccinations and holding concerns about pharmacy management of anaphylaxis and incomplete national immunisation records. The pharmacist wanted to maintain a good relationship with the GP, but the pharmacy continued to offer vaccinations. 

Most midwives were very positive, given the opportunity and convenience specific to the nature of pharmacy versus general practice, and wanting *“more options”*. Some midwives wanted more pharmacies to offer vaccinations.

*“I think the more places that it’s free, you know.”*

M7

One midwife who provided vaccinations herself worried about the local pharmacy being too busy to do vaccinations and where women would wait after vaccinations.

*“Just seems strange to go to a pharmacist for it… but everyone has their own choices so… it’s just somewhere else, but I’ve not heard of many taking that up.”*

M1

Another midwife was also concerned about the busy-ness of pharmacy, and their ability to handle adverse reactions. Noting a client reported feeling rushed and insufficiently informed about the pharmacy-provided vaccination, she recommended general practice to her clients, although she reported receiving a similar complaint from a client about a vaccination from a practice nurse. One midwife preferred using a pharmacy near a medical centre in case an adverse event occurred. 

Most practice nurses were very positive about pharmacists providing maternal vaccinations, although some had qualifications, such as notifying the general practice, pharmacists having appropriate training or having emergency equipment. One practice nurse worried about how an adverse reaction would be managed in a pharmacy, and a practice manager from a Māori general practice preferred Māori-delivered health care for Māori.

*“I think it’s great because it improves access, so long as they’re safe you know and they’re obviously educated to do it, I assume they have oxygen on hand…”*

N1

Three of the four GPs wanted pharmacy notifications on vaccinations provided, with one concerned about reaching targets. One GP in a high-need area noted unmet need with midwives not vaccinating, but worried about anaphylaxis, communication from pharmacy to general practice, the pharmacy being busy, and that the pharmacy was doing it for financial reasons. The other GPs were more positive, with one employee GP noting *“it’s good”* mainly because they were largely lost from general practice in pregnancy (see opportunity below). Although one GP speculated the local pharmacy might not provide vaccinations to maintain their relationship with the medical centre, the two practice owners indicated little financial concern with pharmacists vaccinating:

*“I don’t really have an issue with them doing it…. Does it really matter who gives the vaccination? Not really.…. We’re not in the vaccination game for the money out of it because there is no money in vaccinations…. As long as I’ve got the information, and so that we’re not spending wasted time chasing up someone who’s already had a vaccination…”*

D3 GP and practice owner

### 3.5. Maternal Vaccination Uptake in Pharmacy

Nine women (three Māori/Cook Island Māori and six non-Māori) received one or more maternal vaccinations in pharmacy, sometimes suggested by the midwife or pharmacy, or having seen information in the pharmacy about funded vaccinations. One Māori woman and one Cook Island Māori woman (CM2 and CM4, see case study 1), both living in the same small town, reported only knowing about and having vaccinations whilst pregnant because the pharmacist mentioned it, despite having previous pregnancies. One non-Māori woman only received vaccinations during pregnancy because of the pharmacy:

*“…my midwife hasn’t mentioned it all I don’t think. Then I went to the pharmacy for something else, and the pharmacist just popped her head over and she’s like oh are you pregnant? Just like reminded me that I needed to get the whooping cough the second time round [after a previous pregnancy], coz I actually didn’t know whether I needed to the second time.”*

W11

Women getting maternal vaccinations in pharmacies were largely satisfied with the service, although one reported insufficient privacy waiting for the 20 min post-vaccination period in a public thoroughfare between the pharmacy and other clinics. 

Pharmacists generally reported providing small numbers of maternal vaccinations e.g., one or two per month. One pharmacist working in a low socioeconomic area with high numbers of Māori, Indian and Pacific patients reported informing women about maternal vaccinations but some, particularly non-European did not return for them:

*“… we do a huge amount of dispensing for iodine and folic acid, and, but nowhere near the proportionate amount of vaccines. Most of the vaccinations we do are not of the lower socioeconomic group, they’re of the higher group…”*

P5

Two pharmacists reported relatively high maternal vaccination uptake, one reached nine in one month with a concerted effort from all staff and strong use of promotion (see Proactivity). Another reported providing 10–20 maternal vaccinations in the preceding month, with staff discussing maternal vaccinations and providing leaflets with folic acid and iodine prescriptions, and midwives referring women for the vaccines.

### 3.6. Enablers and Challenges to Recommending and Providing Maternal Vaccinations in Pharmacy

Many enablers and challenges to recommending and providing maternal vaccinations in pharmacy were described (Table 3), with the most important of these discussed throughout this paper.

### 3.7. Themes Emerging from the Interviews

#### 3.7.1. Opportunity—Awareness and Administration

Funded pharmacist-administered maternal vaccinations were considered to provide an opportunity to raise awareness of or provide vaccinations in a convenient way, supplementing existing information and delivery pathways, and with benefits for uptake. 

Most women participants reported hearing about vaccinations during pregnancy from pharmacy staff and/or seeing posters at their pharmacy, even if vaccinated elsewhere.

Many pharmacists reported low awareness amongst pregnant women, or subgroups (e.g., Somalian and Middle Eastern women), supporting the potential for increasing awareness, although some pharmacists thought women’s awareness of maternal vaccinations might be improving. One pharmacist in a lower socioeconomic city area with a high Māori population observed:

*“A lot of women to be honest don’t know about the vaccinations that are recommended in pregnancy…. [or] don’t really know about the timing…”*

P9

Collecting prescriptions for pregnancy supplements from the pharmacy provided a key opportunity for discussing maternal vaccinations, with children’s prescriptions and other visits sometimes providing multiple reminder opportunities. One pharmacist said their staff approached visibly pregnant women regarding vaccination. Some pharmacists noted that mentioning maternal vaccinations allowed an opportunity to address women’s concerns about maternal vaccinations, e.g., needle phobia or safety concerns. 

Midwives were positive that pharmacy provided another opportunity for a woman to become vaccinated, for example, noting they may not go to general practice.

*“So having it at the pharmacy, there could be a much bigger uptake and they’re discussing it with them when they go in to pick up a prescription. So even if their midwife hasn’t discussed it with them and sent them in, they’ve got another opportunity as well.”*

M5

General practice participants also recognised the opportunity for consumer awareness.

*“…if somebody’s in there getting something else and once again opportunistically they could be immunised. So that’s good for the patients, that’s more convenient.”*

N1

Instances were mentioned of some women not engaging with general practice at all or during the pregnancy, or not having a single GP. In one small rural town, where the pharmacies did not deliver vaccines, a practice nurse was very positive about pharmacists delivering vaccinations, indicating she missed approximately 80% of vaccinations that could be given during pregnancy, *“we just don’t see them”.*

Box 1 **Case Study 1: A woman receiving maternal vaccination from pharmacy.**

Case Study 1—MW4

This Cook Island Māori woman living in a high deprivation area first presented to the midwife for this pregnancy at 25 weeks’ gestation. Despite having had her childhood vaccines and reporting her children were vaccinated, she had no maternal vaccinations before, only becoming aware of such vaccinations when the pharmacist informed her about it at 30 weeks’ gestation.

*“I was keen for it because, yeah, anything to help my baby”.*

She had the pertussis vaccine immediately in the pharmacy, but influenza vaccine was unavailable (for summer). While nervous about getting the vaccine, *“just because it’s different ay, to come into a pharmacy and getting that stuff done”*, she reported feeling very comfortable with the pharmacist administration. As *“a walk in,”* she found pharmacy *“a lot easier”* for the vaccination, presumably than general practice where vaccinations are usually provided, and free administration was an important enabler for her.

#### 3.7.2. Convenience and Accessibility, Just ‘Pop in’

Convenience and accessibility both arose frequently. Many participants across all groups appreciated that pharmacies required no appointment, would be likely to have a shorter wait before the vaccination, and typically had longer opening hours than general practice. 

Women who used the pharmacy for the vaccination did so for convenience, noting two reasons: not requiring an appointment; and expecting less waiting time than in general practice. Several observed that having other children to look after or work commitments meant the pharmacy accessibility was helpful.

*“… through the GP it’s a bit of a pain to make an appointment and go in and with a pharmacy you basically just walk in. Which is quite easy.”*

W18

Conversely, one woman contacted several pharmacies who did not provide vaccinations or had no vaccinating pharmacist available on a Saturday before finding one that suited her timing. One woman used her general practice, being *“easier”* than pharmacy because she was there for blood tests.

One pharmacist reported doing most maternal vaccinations on Saturdays, but another reported insufficient staffing to offer vaccinations on Saturdays.

Some midwives in high-need areas and with mainly Māori clients outlined challenges to women in accessing general practice including transport/money for petrol, difficulty booking appointments, and chaotic lives and appreciated pharmacy having no appointments. One Māori midwife highlighted the challenges some clients had with booking and keeping appointments:

*“So my typical clients … they’re not booking an appointment at the GP. A lot of people I need to make three appointments to see them once.”*

M5

#### 3.7.3. Proactivity

Linked with the opportunity theme, some pharmacists were particularly proactive in raising awareness of maternal vaccinations. 

The main proactivity in the pharmacies was in having posters displayed, giving out pamphlets or using dispensary labels to provide a brief message about maternal vaccinations (particularly with prescriptions for folic acid or iodine in pregnancy), or providing verbal messages about vaccinations. Some women participants had noticed posters in the pharmacy about maternal vaccinations, and these posters were obvious in some pharmacies when doing the interviews, e.g., on the door as the pharmacy was entered, in the pharmacy window, in the consultation room and on the wall or door outside the consultation room.

Some pharmacists informed local midwives of their service and gave them leaflets for women or posters about maternal vaccinations. A few pharmacist participants involved their non-pharmacist staff in informing pregnant women. 

One pharmacist said that if a woman was not ready to have a vaccination after a discussion, she would diary a call back to remind her about the vaccine. Other pharmacists had no call back or text system but saw merits in using it for pertussis vaccine given many women were not yet in the 28–38-week window when the pharmacist discussed vaccination with them. However, pharmacists were concerned about possible miscarriage before the text, and so would seek the woman’s permission, limit contact, and word the text carefully.

Box 2 **Case Study 2: Proactive pharmacy.**

Case study 2—P7

One pharmacy with two keen vaccinating pharmacists was especially proactive. Participant P7 described herself as *“passionate”* about vaccinations, having been involved in a vaccination project at under-graduate level. Together with pharmacy staff, they made the most of the resources available, having posters in the pharmacy and consultation room, staff wearing maternal vaccination promoting t-shirts on certain days, distributing pamphlets about maternal vaccinations, and posting information and video clips on the pharmacy Facebook page. To increase staff knowledge on maternal vaccinations, they used the one-page information sheet sent from the researchers to all pharmacies, discussed them at staff meetings, and encouraged front-line staff to complete the two quizzes on maternal vaccination.

They informed their local midwives that they provided vaccinations and gave midwives leaflets for their pregnant women regarding vaccinations, resulting in midwives referring women to them for the service.

On seeing prescriptions for folic acid, iodine or iron tablets, or pregnant women in the pharmacy for other reasons, a staff member would discuss vaccinations and provide a leaflet.

Tdap vaccine was kept in stock to ensure it was always available.

This pharmacy set a target of administering 10 maternal vaccinations for one particular month (versus two or so per month usually), encouraging staff to remember to mention it to pregnant women and posting more on Facebook. They reached nine maternal vaccinations that month, largely driven by the staff raising awareness. This high rate was not sustained though, partly because women who seemed interested did not return for it.

#### 3.7.4. Missed Opportunity

Although some pharmacies were proactive, missed opportunity also existed, particularly where individual pharmacies did not offer vaccinations. 

Two women participants received no influenza vaccine, reportedly told by pharmacists that it was the wrong time of the year. 

One NZ European woman received no vaccinations despite being “pro-vaccination” and knowing about maternal vaccinations from verbal messaging and posters in the pharmacy and her midwife). This participant had personal and health struggles, and an older child and full-time work, giving her little capacity to consider vaccinations. Had she been offered vaccinations in the pharmacy while waiting for a prescription she *“definitely”* would have had it. *“You don’t have to think about it, you don’t have to go anywhere. It’s done.”*

The opportunity to offer or discuss maternal vaccinations was reduced when a pregnant woman did not collect prescriptions from the pharmacy. Midwife-sent prescriptions were often uncollected in one pharmacy in a high-needs area. When a third-party collected the prescription, pharmacists’ privacy concerns meant vaccination information was not given. One pharmacist only discussed vaccinations during pregnancy when patients were obviously pregnant, not raising it when first dispensing pregnancy supplements:

*“…because you don’t know whether they’re pleased to be pregnant, whether they’re in a relationship or etc. it’s a bit dodgy to say ‘Oh I see you’re pregnant, you need to make sure you get your flu vaccine and your free whooping cough vaccine’ because you don’t know what state of mind they’re in and that’s the dilemma I have at this stage.”*

P5

This pharmacist also noted many women were not getting iodine prescriptions later in pregnancy (the first prescriptions lasts three months), also limiting the opportunity to discuss vaccinations. 

Some pharmacists, despite enjoying providing vaccinations, reported that being busy with prescriptions reduced the opportunities to mention vaccinations. One time-poor pharmacist in a high-need area put labels on dispensed pregnancy supplements informing about vaccinations in lieu of a conversation, but reported administering no vaccinations to pregnant women, indicating that this strategy was ineffective. A midwife in that community reported low literacy was common, potentially limiting the effect of a written label without discussion. 

One vaccinating pharmacist had not used pregnancy supplement prescriptions as a prompt to raise vaccinations, but thought it was a good idea. Most pharmacists reported that non-vaccinating pharmacy staff did not identify and refer pregnant women for a discussion about vaccinations, due to insufficient knowledge or vaccinations not being top of mind.

Some pharmacists did not inform local midwives that they administered maternal vaccinations. Such contact would raise the awareness of this service in pharmacies, provide an opportunity to answer questions (e.g., about costs), and help midwives signpost to pharmacies offering the service. 

Some communities had no pharmacies providing vaccinations, including a remote area in which two midwives wanted the pharmacy to offer vaccinations to aid access given residents’ challenges travelling to larger centres or making appointments in general practice. One midwife appreciated that one pharmacy in her small town provided vaccinations, but worried that women attending non-vaccinating pharmacies for prescriptions in pregnancy would not receive messages about vaccinations.

#### 3.7.5. Time and Waiting

Time often arose throughout the interviews, with saving time as a benefit of pharmacy delivery of maternal vaccinations, and insufficient time limiting opportunistic discussions about vaccinations in pharmacy. Convenience and accessibility of pharmacies, including long opening hours, helped women with limited time (e.g., with other children or work commitments). Some women and midwives expected a shorter pre-vaccine wait in pharmacy than general practice. 

Pharmacists reported usually managing time to provide vaccinations when requested, or help address concerns such as needle phobia, without needing to arrange appointments. However, a few pharmacists discussed time pressures, limiting their patient contact, and proactivity with vaccinations. Waiting for training courses frustrated a pharmacy owner, limiting the number of staff able to vaccinate.

#### 3.7.6. Trust and Communication

Trust, relationships and communication arose throughout the interviews. For women and some health professionals, having a relationship with a pharmacy and knowing pharmacists had appropriate training to provide vaccinations aided trust.

*“I wouldn’t just rock up to a random pharmacy, …. I’d want to feel like I trusted the place...”*

MW1

Unfamiliarity with pharmacies providing vaccinations caused some hesitancy about receiving a vaccination from there (see Box 1 and Box 2 case study). 

Most general practice staff indicated trust in pharmacists vaccinating. A minority of midwives and general practice staff worried about management of adverse reactions in pharmacy, and one employee doctor distrusted pharmacists’ motives to offer the service. 

Trust and communication also related to cultural aspects. A Māori general practice manager preferred Māori to deliver health services for Māori rather than pharmacy. A pharmacist worried about a cultural divide with their Māori patients, but could not attract Māori staff. One Māori woman was initially hesitant in being vaccinated in pharmacy, but was very comfortable with her experience, although her pharmacist was also Māori, which may have helped. Pharmacists spoke of challenges with women with limited English. Another pharmacist noted an ability to connect with different ethnicities as a non-European himself, feeling that engendered trust. 

Midwives often advised women of maternal vaccinations availability in pharmacy and general practice. General practice staff reported never communicating pharmacy availability as an option, but two pharmacists reported advising patients of general practice as another option for maternal vaccination administration.

## 4. Discussion

This is the first qualitative study of women and different health care providers exploring provision of funded maternal vaccinations through pharmacy. We interviewed 53 participants, comprising 18 women who were pregnant or recently pregnant, 12 pharmacists, 11 midwives and 12 general practice staff. We found strong positivity towards funding maternal vaccinations in pharmacy. Only a small minority of health professionals expressed reservations which were primarily focused on procedural issues. Funding these vaccinations through pharmacy appeared to address some barriers to access, and, in some cases, aided awareness and uptake.

Most participants (women and health care professionals) saw benefits in extending the current funding of maternal vaccinations to pharmacy, given challenges in the existing care model and an opportunity to raise awareness. Providing another government-funded delivery option outside of primary care with convenience and access, and particularly the mechanism of unbooked opportunistic immunisation, was considered likely to increase uptake. Some women only received a vaccination during pregnancy because of their pharmacist, having been unaware of it previously. Women receiving vaccinations in pharmacy largely had positive experiences, although one woman experienced difficulties finding a vaccinating pharmacist. 

Pertussis vaccination during pregnancy is already government-funded through general practice, with low uptake. Extending funding to pharmacy is likely to increase the awareness and uptake and should have a similar cost-benefit to the current programme, assuming the vaccine cost and administration costs remain the same. With approximately 60,000 births each year, the cost of this programme extension to pharmacy should not be excessive but would depend on uptake. As the government is committed to funding vaccine uptake in general practice, extending funding to pharmacy may be more cost-effective than the cost of trying to increase uptake in general practice. 

A minority of general practice staff and midwives worried about pharmacies busy-ness or ability to manage adverse effects. Pharmacists did not express such concerns for managing vaccine administration, possibly because they felt prepared with training and emergency equipment in the unlikely event of a serious adverse effect. In New Zealand, pharmacists have insurance and can be disciplined in case of an error, but medical misadventure is usually covered by the Accident Compensation Corporation regardless of health practitioner. 

General practice staff wanted notifications about vaccinations administered to their patients in pharmacy. Communication between pharmacy and midwives and general practice appeared to help raise their awareness of vaccinations in pharmacy, and some midwives suggested pharmacy-delivery for maternal vaccinations to their clients.

Some missed opportunities appeared, e.g., pharmacies providing no vaccines or having no promotional material, pharmacies not offering vaccines at the weekend, or pharmacists being too busy to mention vaccinations to pregnant women. Providing leaflets or stickers about maternal vaccinations instead might be ignored by those with low health literacy and provides no opportunity to address any concerns. Pharmacists varied in their proactivity, with one pharmacy using all staff to increase their maternal vaccination uptake. 

Widening vaccination administration to pharmacy is associated with increased uptake [32,33]. A pertussis control workshop in NZ recommended pharmacists promote immunisation when providing folic acid and pregnancy tests to pregnant women to aid uptake [22], and a qualitative NZ study found women supported availability of funded pertussis vaccine in pharmacy, considering it would raise awareness and usage in pregnant women [21]. The current research provides additional evidence that funded pharmacy availability of pertussis and influenza vaccinations during pregnancy did raise awareness and uptake in some of the women interviewed. 

Research conducted in the United States in 2010, found vaccinating pharmacists less positive about giving influenza vaccination during pregnancy than to the general public, with vaccine safety concerns raised [44]. While a non-vaccinator pharmacist voiced concern, our pharmacist vaccinator participants did not, possibly because maternal vaccinations were more established than in the earlier study, or pharmacists were well equipped from the education on vaccinations we provided. 

Vaccinator pharmacists and recipients of pharmacist-administered vaccines were generally positive about the service, similar to other research [45,46,47] which also found convenience an important driver. However, unfamiliarity with vaccines in pharmacy caused some women hesitancy about using the pharmacy. Having more vaccines available and funded in pharmacy and all pharmacies providing vaccinations would help normalise the service. 

The support of funded maternal vaccinations in pharmacy by most general practice staff (including two general practice owners) differs from previous negativity reported from general practice in NZ regarding pharmacists vaccinating [48,49], and experiences or concerns three pharmacists reported in this study. Participants’ concerns about the gap between general practice and midwife care, and the relatively few maternal vaccinations given in general practice may have helped this support. Other vaccine providers may gradually accept pharmacists vaccinating as others have suggested [50]. Interviewing more nurses than doctors may have influenced our findings; in Canada, nurses accepted pharmacist vaccinators more than doctors [51]. Some pharmacists may need encouragement to help overcome perceived or actual concerns from other providers.

This research suggests that funding maternal vaccinations in pharmacy is likely to overcome some barriers to uptake through addressing gaps in awareness and increasing availability. Two women in late pregnancy previously unaware of vaccinations who the pharmacist informed and vaccinated, were in a high-need area, and both were Māori, a group disproportionately affected by pertussis during infancy [52], suggesting that this policy could help equity of access. Our interviews revealed that some pregnant women need as much help as possible to get recommended vaccinations. Other research which observed the many demands of pregnancy recommended increasing vaccine availability to help those more passive [53]. Pharmacist involvement in vaccination aids uptake [32,54], but the effect on maternal vaccinations has received little attention. Research from NZ, conducted in 2014 [21], found many women unaware of maternal vaccinations and none being informed by the pharmacist, whereas we found most women had received information from the pharmacy about vaccinations. In one pharmacy in a high-needs area, written information appeared of little value in increasing uptake, and other maternal vaccination research found many women prefer verbal communication to being given a leaflet [55]. However, time for this can be a challenge as we found and others have discussed [56], and better use of non-pharmacist staff could help. 

Educating pharmacy technicians and pharmacy assistants about vaccination could increase pharmacy proactivity. Increasing vaccinator staff should also help, using intern pharmacists (pre-registration), and pharmacy technicians would help raise awareness, help the pharmacy be more proactive about maternal vaccinations, and allow vaccinations throughout all opening hours. Educating pharmacy technicians about vaccinations helped them raise vaccinations with consumers in a US study [50], and pharmacy technicians as immunisers occurs in some jurisdictions [49,57,58]. Text reminders from the pharmacy might also aid uptake, as found with maternal vaccinations from other health care providers [59], but would need sensitivity, and should not replace verbal conversations.

The applicability of this research to other countries may depend on the model of care and uptake. Gaps between providers and/or limited awareness and uptake of maternal vaccinations may occur elsewhere [53,55,60], and more convenient and accessible options, and other health care providers to raise awareness may help those with the greatest needs. 

For this initiative to work optimally, funded provision from all pharmacies, particularly in high-need areas, including remote areas, would help. Barriers to pharmacies offering vaccinations include sufficient funding, the need for training, and staff time [49,54,61,62]. In NZ, only influenza vaccination for specific patient groups is funded in pharmacies nationally. Funding more vaccinations in pharmacy would increase viability of the service in low-income areas with no private vaccine market, raise the profile of vaccines for pharmacy staff (making them more top-of-mind) and increase consumer familiarity with pharmacy-delivery of vaccines. Furthermore, more funded vaccines could reduce challenges of minimum orders.

Quantitative data are needed to ascertain how funding maternal vaccination administration in pharmacy helps overall uptake, work that our team is undertaking currently.

### Strengths and Limitations

We conducted 53 interviews that explored funded maternal vaccinations in pharmacy from the perspectives of 18 women and 12 pharmacists, 11 midwives and 12 general practice staff. Previously published maternal vaccination qualitative research has typically excluded pharmacists [55]. Wilson et al. [55] similarly used a high number of interviews to explore strategies to improve maternal vaccine uptake, with a different balance of 40 women, six GPs, two practice nurses and two midwives, however pharmacy-delivered vaccination appeared not to arise. The higher number of women in this London study allowed a greater range of ethnicities and more vaccine-hesitant participants than in our study. 

Qualitative research exploring pharmacy-delivered vaccinations has typically been limited to consumers’ [21,63,64,65] and/or pharmacists’ views [47,50,66,67], while a strength of this study was the inclusion of other health care provider groups, pharmacists and consumers. 

We chose our sample purposively to ensure that a wide range of experiences and views were captured. As indigenous people are disproportionately affected by vaccine-preventable diseases [68], we particularly focused on Māori and high-need areas where the learnings could be greatest. We used a Māori interviewer for Māori women. 

Most women were recruited in pharmacy, and so women not attending pharmacies were effectively excluded. Some early interviews with Māori women were informative but short, but later interviews provided more depth. We interviewed few young women, with only one under 20 years, an age group associated with low maternal vaccination uptake [69]. We focused on Māori particularly, but not other non-European ethnicities in our sampling of the women participants. 

Data saturation was not sought but rather breadth, with a range of health care providers and women interviewed. 

We deliberately focused much of the sample on high-need populations, and conducted a large number of interviews. However, quantitative research is needed to show exactly what effect funding of pregnancy vaccinations in pharmacy has on uptake across all pregnancies and especially those in high-need groups. A cost analysis could also be helpful. 

## 5. Conclusions

The acceptance of immunisations occurring outside primary care in pharmacies means this is a valid way to increase uptake and prevent disease. Government-funded maternal vaccinations in pharmacy appear to address some barriers to uptake by increasing awareness and providing a more accessible option for administration that needs no appointment. This service appears to be useful, including in high-need communities, and was generally well accepted by women, pharmacists, midwives, and general practice staff. Maximising the opportunity of pharmacy to raise awareness and uptake requires all pharmacies to provide vaccinations, more vaccinators in pharmacy, and greater awareness and proactivity in front-line staff. 

## Figures and Tables

**Table 1:** Characteristics of the enrolled and interviewed women (*n* = 18).

Variable	Māori or Pacific Women *n* = 10	Non-Māori Women *n* = 8
Ethnicity	9 Māori and 1 Cook Island Māori.	4 NZ European; 3 South African European; 1 Chinese (born in China).
Rural/urban	6 lived in rural dwellings, 4 in urban.	2 lived in rural dwellings, 6 in urban.
Age	Aged 18–31 years; 5 were ≤25 years.	Aged 23–37 years; 1 was ≤25 years.
Number of weeks pregnant or age of infant at the time of the interview	1 was 22 weeks pregnant and 4 were 31–39 weeks pregnant. 5 had infants aged 5 weeks to 4 months old.	3 women were 34–36 weeks pregnant. 4 had infants 1 week to 4.5 months old, and one had a 12-month-old infant.
Number of children	2 women had no other children, 3 had 1 other child, 1 had 2 other children, 1 had 3 other children, and 3 had 4 other children.	3 women had no other children; 4 had 1 other child, and 1 had 2 other children.
Lead maternity carer	All used a midwife.	All used a midwife.
First presentation to their lead maternity carer	5 women first saw their midwife at 4–7 weeks’ gestation, 2 at 12–15 weeks, and 3 at 25–27 weeks.	All women had first presented to the midwife at 4–10 weeks’ gestation.
No. who received maternal vaccinations	2.	1.
Vaccinations received during this pregnancy (or planned to be received)	3 received both vaccinations, 1 planned to receive both vaccinations, 4 received Tdap but not influenza vaccine.	2 received both vaccinations, 4 received pertussis but not influenza vaccine and 1 received influenza but not Tdap vaccine.
Location of vaccinations given during pregnancy	Vaccinations given at the pharmacy (*n* = 3), general practice (*n* = 3 and 1 intending to) and hospital (*n* = 1).	Vaccinations given at the pharmacy (*n* = 6) and general practice (*n* = 1).

**Table 2:** Characteristics of the enrolled and interviewed health care practitioners (*n* = 35).

Variable	Community Pharmacists (*n* = 12)	Midwives (*n* = 11)	General Practice Staff (*n* = 12)
Staff mix	10 (including 3 pharmacy owners) were trained vaccinators providing vaccinations. 2 were pharmacy owners in pharmacies not providing vaccinations (1 owned other pharmacies where vaccinations occurred and was vaccination trained).	1 worked at a hospital, 10 worked as independent midwives—3 of whom had recent or current hospital or district health board experience.	4 general practitioners (2 practice owners), 7 practice nurses, 1 practice manager.
Rural/urban	7 rural, 5 urban.	7 rural, 4 urban.	8 rural, 4 urban.
Practice details	6 worked in high-need areas, 2 were in higher socioeconomic areas, the rest had mixed socioeconomic clientele.	2 described their area served as high socioeconomic, the rest were in low socioeconomic or mixed socioeconomic areas. The Māori midwives tended to have mainly Māori clients.	All worked in practices with mostly high deprivation, with mixed high and low deprivation, and/or high Māori patient load. 2 worked at a Māori health care provider.
Work hours	11 worked full time and 1 worked part-time.	All worked full-time or close to it, with 2 working about 60 h per week or more.	7 were full-time, 4 part-time and 1 unknown.
Ethnicity	6 identified as NZ European, 3 as Chinese/Asian), 1 as Māori, 1 as Fiji Indian and 1 as Middle Eastern.	5 identified as Māori or part Māori, 4 as NZ European, 1 as British, and 1 as Asian.	8 identified as NZ European, 1 was South Asian, 3 were Māori or NZ European/Māori.
Experience	4 had 1–4 years’ experience, 5 had 9–18 years’ experience and 3 had 30–40 years’ experience.	3 had 1–5 years’ experience, 4 had 7–15 years and 4 had 20–30 years’ experience.	2 had 2–5 years’ experience, 4 had 9–16 years’ experience, and 6 had 25–31 years’ experience.
Gender	8 female and 4 male.	All female.	All female.

**Table 3:** Enablers and challenges for pharmacy in providing vaccinations or information about vaccinations during pregnancy.

Enablers	Challenges
Pregnant women visiting the pharmacy for prescriptions and other reasons. Pharmacists enjoying providing vaccinations. Staff seeing vaccinations as an important role for pharmacy. Sufficient qualified staff at all times. Informing midwives of their service and having them recommend the pharmacy for maternal vaccinations. Awareness in front-line staff referring pregnant women to the pharmacist. Using pamphlets, posters, Facebook posts, t-shirts. Having a relationship with the woman or being trusted. Setting a target number of vaccinations for the pharmacy. Nice waiting area, good consultation room. Familiarity from doing many influenza vaccines. Consumer self-completion of the check list. Open extended hours, e.g., 7 days per week.	Busy with dispensing. Insufficient staff qualified in vaccinating or first aid. Staff unaware of or not thinking about maternal vaccinations. Concentrating on other services. With few vaccinations administered outside of influenza vaccination time the pharmacist can feel rusty and have vaccination less top-of-mind. Finding locums who are trained vaccinators. New pharmacists need training. Women without time to wait after a vaccine (and hence delay their vaccinations). Maintaining relationships with or negative feedback from a medical practice. Insufficient fridge size. Located within or alongside a medical centre. Woman is not obviously pregnant. Prescription is collected by someone else. Public unfamiliarity with pharmacists vaccinating. Cost of training pharmacists. Buying stock—minimum orders or a fee of NZ$45 (US$28.50). Delivery issues—cancelled order/data logger problem.
